# Treatment of MDA5-positive dermatomyositis complicated by gangrenous cholecystitis with tofacitinib

**DOI:** 10.1186/s40001-022-00693-0

**Published:** 2022-05-15

**Authors:** Man Luo, Long Chen, Huan He, Fang He

**Affiliations:** Suining Central Hospital, 127 Desheng West Road, Chuanshan, Suining, 629000 Sichuan China

**Keywords:** Dermatomyositis, Tofacitinib, Anti-MDA5 antibody, Gangrenous cholecystitis

## Abstract

**Background:**

Dermatomyositis is a rare idiopathic inflammatory disease with diverse presentations that can have varying degrees of cutaneous and systemic involvement. This phenotypic heterogeneity makes DM a therapeutic challenge. Some therapeutic drugs, such as hormones and immunosuppressants, have poor therapeutic effects. In recent years, tofacitinib has been reported to be effective in the treatment of dermatomyositis.

**Case presentation:**

We report a case of anti-MDA5 antibody-positive dermatomyositis that was relieved after treatment with tofacitinib, during which gallbladder gangrene and suppurative cholecystitis occurred. After cholecystectomy, we continued to use tofacitinib and achieved a good therapeutic effect.

**Conclusions:**

Tofacitinib is effective in the treatment of anti-MDA5 antibody-positive dermatomyositis, but the risk of infection is increased. It can still be used after infection control. Close follow-up should be performed during the use of tofacitinib.

## Background

Dermatomyositis (DM) is an idiopathic inflammatory myopathy (IIM), and a variety of myositis-related antibodies can be detected. In particular, MDA5-positive dermatomyositis is characterized by interstitial lung disease, subcutaneous calcification, myalgia, skin involvement and vascular lesions [1, 2]. Some forms of DM cannot be completely relieved with or even relapse on therapeutic drugs, including glucocorticoids and traditional immunosuppressants [3]. In recent years, there have been many reports that the Janus kinase (JAK) inhibitor tofacitinib is effective in the treatment of DM, but increases the risks of infection and thrombosis [4]. We report a case of anti-MDA5 antibody-positive dermatomyositis that was relieved after treatment with tofacitinib, during which gallbladder gangrene and suppurative cholecystitis occurred. There are no reports of similar cases at present.

## Case presentation

**Fig.1:**
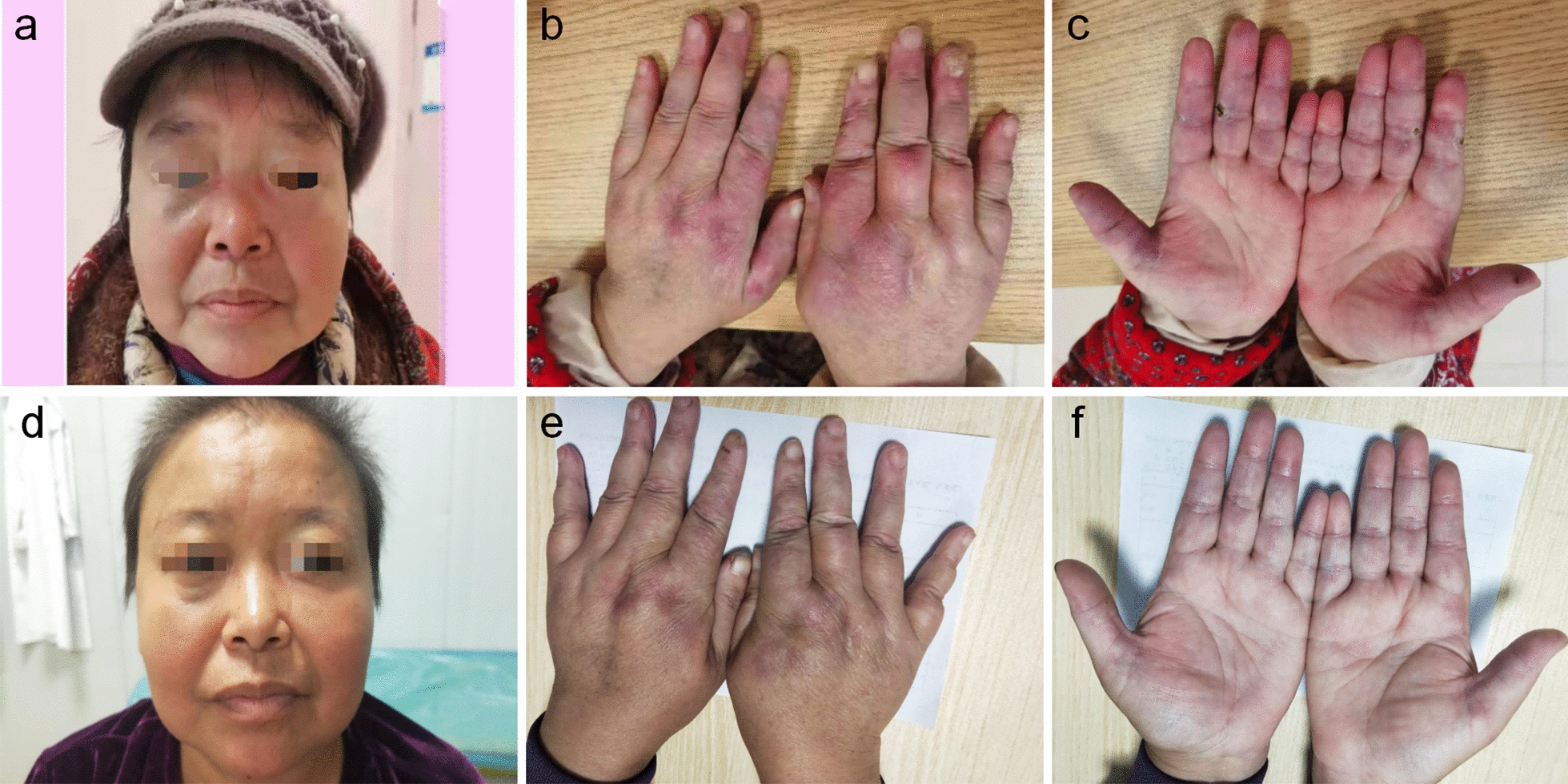
Clinical course. Skin lesions on face (**a**), palmar and opisthenar surface of hand with erythema (**b**) and ulcerations (**c**) before and after (**d**, **e**, **f**) treatment with tofacitinib for 6 months

**Fig.2:**
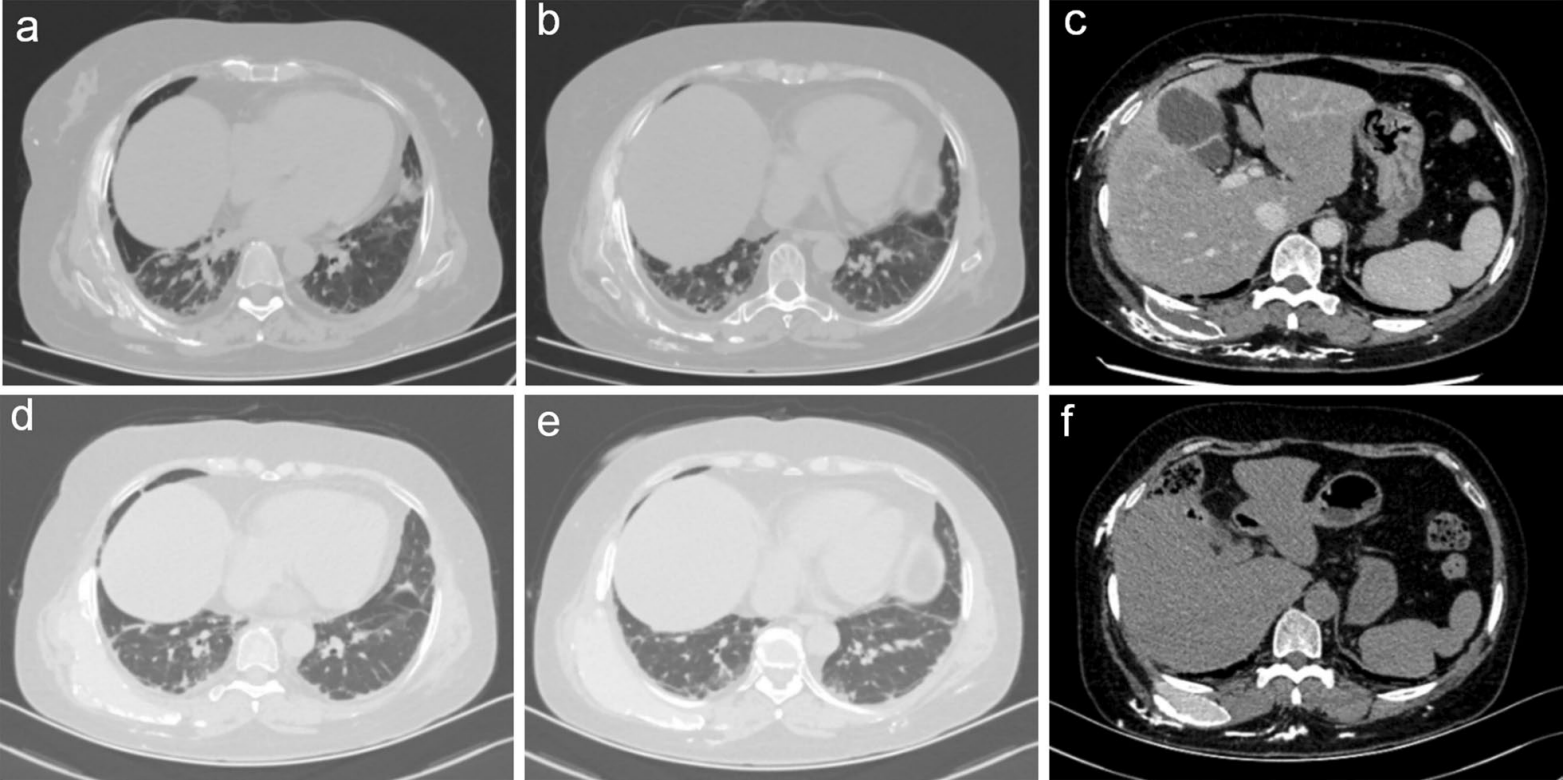
Chest computed tomography HRCT of the lung before (**a**, **b**, **c**) and after (**d**, **e**, **f**) treatment with tofacitinib for 6 months

More than 2 years ago, a 56-year-old woman had a skin rash on the face (Fig. 1a), eyelids, neck, chest and fingers of both hands (Fig. 1b) and Raynaud's phenomenon in both hands, accompanied by finger ulcers (Fig. 1c), limb weakness, myalgia, dysphagia, joint pain, cough, airway constriction, intermittent fever, and palpable nodules on the chest wall, hip and left thigh. Her 6-min walk test result was 321 m. The CK, ALT and AST levels were normal; she was anti-MDA5 IgG positive; the Ro52 level was 280.26 RU/mL; the ANA titre was 1:1000; and the pattern was of the nuclear granular type. Chest computed tomography showed chronic inflammation of the lungs with multiple interstitial changes (Fig. 2a, b) and multiple subcutaneous calcifications (Fig. 2c). Pulmonary function indicated decreased DLco (22%). Electromyography showed that the time limit and amplitude of light muscle contraction were normal and that the polyphase potential was increased. The diagnosis met the 2017 EULAR/ACR classification standard [5]. Prednisone acetate combined with matimecophenol ester or cyclosporine and cyclophosphamide produced poor therapeutic effects. The patient had her skin rash, ulcers and dyspnoea relieved after approximately one month of treatment with prednisone (15 mg qd) and tofacitinib (5 mg qd). However, chills and fever with a maximum temperature of 40 °C occurred on February 25, 2021, and the patient had epigastric pain and tenderness, with a positive Murphy test. Abdominal colour Doppler ultrasound indicated cholecystitis. CT of the upper abdomen showed that the gallbladder was slightly enlarged, and the internal density was not uniform; the gallbladder wall was suspected to have uneven thickening and local nodular changes, and the border of the gallbladder was blurred. After treatment with prednisone (15 mg qd) and piperacillin tazobactam for 3 days, the patient still had fever, abdominal pain and a leukocyte count of 16.6 × 10^9^/L. The treatment regimen was adjusted to imipenem/cilastatin to eliminate any infection for 7 days, until the patient had no fever. Cholecystectomy was performed on March 12, 2021, and a frozen section of the bottom of the gallbladder was sent for examination. A few tissues had acute and chronic suppurative inflammation with necrosis. Postoperative examination indicated acute gangrenous cholecystitis of the gallbladder. The patient resumed prednisone (15 mg qd) and tofacitinib (5 mg qd) treatment starting on March 30, 2021. Five months later, the rash on both hands (Fig. 1e) and the face had subsided (Fig. 1d), the ulcers on both hands had completely healed (Fig. 1f), and the range of HRCT interstitial changes in the lungs (Fig. 2d, e) was significantly decreased. Her 6-min walk test result was 506 m, and her DLco (Table 1) improved from severely impaired to mildly impaired. Subcutaneous calcification (Fig. 2f) was reduced.

**Table 1:** Changes of pulmonary function before and after treatment with tofacitinib for 6 months

Variable	Before		After	
	Absolute	% of predicted	Absolute	% of predicted
FVC (L)	1.37	49	1.21	44
TLC (L)	1.09	27	1.05	26
FEV1 (L)	1.08	51	1.05	51
FEV1/FVCmix (%)	79	104	87	115
Peak flow (L/s)	3.44	64	3.31	62
DLCOcSB (mmol/min/kPa)	4.2	22	12.9	65
TLCOc/VA (mmol/min/kPa/L)	3.85	95	12.29	309

## Discussion and conclusions

Dermatomyositis is a rare inflammatory disease with characteristic cutaneous findings and varying levels of systemic involvement. Both immune and nonimmune mechanisms are involved in the pathogenesis of DM. Myositis-specific antibodies can be detected in the serum, and these can help with prognostication, alerting the clinician of systemic manifestations that are more likely in the patient. For example, some anti-MDA5 antibody-positive DM patients achieve only poor therapeutic effects with hormones and immunosuppressants [6]. In recent years, tofacitinib has been reported to be effective in the treatment of DM. Tofacitinib is a relatively nonspecific JAK-i that affects the phosphorylation of different target STATs (including STAT1 and STAT3) and inhibits a variety of proinflammatory cytokines. The JAK/STAT signalling pathway is activated by IFNs, leading to the transcription of IFN-γ-stimulated genes (ISGs), including MDA5. Tofacitinib inhibits this pathway, decreasing MDA5 expression and activation. Tofacitinib has a good therapeutic effect on MDA5-positive DM [7].

We report a case of MDA5-positive dermatomyositis without remission after hormone and traditional immunosuppressive therapy. Therefore, we treated the patient with prednisone and tofacitinib. After 1 month, all of the patient’s symptoms were relieved; however, severe cholecystitis and gangrene occurred. Burmester et al. [8] and Fleischmann et al. [9] reported the risk of upper respiratory tract infection after treatment of dermatomyositis with tofacitinib [10]. Acute gangrenous cholecystitis occurred in our patient. In addition to inflammation, gallbladder circulation disorder is a main cause of gangrenous cholecystitis, leading to bleeding and gallbladder tissue necrosis [11]. Patients with dermatomyositis can have vasculitis, leading to tissue ischaemia and necrosis. There are few reports of similar cases. There is an increased risk of infection during the treatment of connective tissue diseases with tofacitinib. Tofacitinib was used again 2 weeks after the infected tissue was removed. After 5 months, the patient's condition was controlled, and no infection occurred.

In recent years, there have been many reports on the treatment of autoimmune diseases and infectious diseases with tofacitinib. Lee et al. [12] reported on systemic lupus erythematosus with cholecystitis as the first manifestation. Tofacitinib is effective in the treatment of rheumatoid arthritis, ankylosing spondylitis, psoriatic arthritis, Behcet's disease, systemic vasculitis and other diseases [13]. Zhu et al. [14] reported on tofacitinib treatment of refractory cutaneous leukocytoclastic vasculitis. In infectious diseases, Tatiana [15] reported that tofacitinib was effective in 320 patients with COVID-19; this study evaluated a prospective observational series. Tong found that tofacitinib reduced death or respiratory failure at 28 days in patients hospitalized with COVID-19 pneumonia [16].

However, there are increased risks of thrombosis and infection, during the treatment of connective tissue diseases with tofacitinib [4].

In conclusion, tofacitinib is effective in the treatment of MDA5-positive dermatomyositis. However, the risk of infection, which leads to dysfunction in important organs, is increased. It is still effective to use tofacitinib again after the infection is controlled. Therefore, close follow-up should be performed during the use of tofacitinib.

## Data Availability

Not applicable.
